# Cholesterol-Lowering Activity of the Major Polyphenols in Grape Seed

**DOI:** 10.3390/molecules16065054

**Published:** 2011-06-17

**Authors:** Sathaporn Ngamukote, Kittana Mäkynen, Thavaree Thilawech, Sirichai Adisakwattana

**Affiliations:** 1. The Medical Food Research and Development Center, Department of Transfusion Medicine, Faculty of Allied Health Sciences, Chulalongkorn University, Bangkok, Thailand; 2. Program in Nutrition and Dietetics, Faculty of Allied Health Sciences, Chulalongkorn University, Bangkok, Thailand; 3. The Research Group of Herbal Medicine for Prevention and Therapeutic of Metabolic Diseases, Chulalongkorn University, Bangkok, Thailand; E-Mails: amppam10@gmail.com (S.N.); kittana.chanda@gmail.com (K.M.); p_punch_tt@hotmail.com (T.T.)

**Keywords:** polyphenols, grape seed, mechanism pancreatic cholesterol esterase, cholesterol micelles, bile acid

## Abstract

The major polyphenols in grape seed have been shown to have beneficial health effects in the prevention of dyslipidemia and cardiovascular diseases. In this present study, we investigated the cholesterol-lowering activity of three major polyphenolic compounds found in grape seed. The results showed that gallic acid, catechin, and epicatechin significantly inhibited pancreatic cholesterol esterase in a concentration-dependent manner. Moreover, they bound to taurocholic acid, taurodeoxycholic acid, and glycodeoxycholic acid at levels ranging from 38.6% to 28.2%. At the concentration of 0.2 mg/mL, gallic acid, catechin, and epicatechin reduced the formation of cholesterol micelles 27.26 ± 2.17%, 11.88 ± 0.75%, and 19.49 ± 3.71%, respectively. These findings clearly demonstrate that three major polyphenolic compounds present in a particular grape seed have cholesterol-lowering activity by inhibiting pancreatic cholesterol esterase, binding of bile acids, and reducing solubility of cholesterol in micelles which may result in delayed cholesterol absorption.

## 1. Introduction

Recently, the consumption of dietary polyphenols has been associated with a reduced risk of developing chronic conditions such as diabetes mellitus and cardiovascular diseases [1,2]. Grape seeds are considered as a good source of polyphenolic compounds which have been shown to have various beneficial pharmacological effects, including anti-hyperlipidemic [3], anti-inflammatory [4], and antibacterial activities [5]. Polyphenols in grape seed are mainly gallic acid and the monomeric flavan-3-ols catechin and epicatechin [6,7,8]. These polyphenolic compounds have generated a remarkable level of interest based on their protective effects in dyslipidemia and cardiovascular diseases. For example, gallic acid shows evidence of suppression of a high-fat diet-induced dyslipidemia, hepatosteatosis and oxidative stress in rats [9]. A recent study has clearly revealed that polyphenols, mainly catechins, have anti-oxidative properties by the inhibition of Low-Density Lipoproteins (LDL) oxidation [10,11]. Furthermore, epicatechin also protects endothelial cells against oxidized LDL by scavenging free radicals and maintaining nitric oxide synthase [12]. Although the anti-dyslipidemic activities of the major polyphenols in grape seed have been well investigated, studies regarding their effects on cholesterol digestion and absorption have not been undertaken. Therefore, the aim of the study was to evaluate the effects of major polyphenols in grape seed – gallic acid, catechin, and epicatechin – on the inhibition of pancreatic cholesterol esterase, bile acid-binding capacity, and the solubility of cholesterol micellization.

## 2. Results and Discussion

### 2.1. Inhibition of Pancreatic Cholesterol Esterase

**Figure 1:**
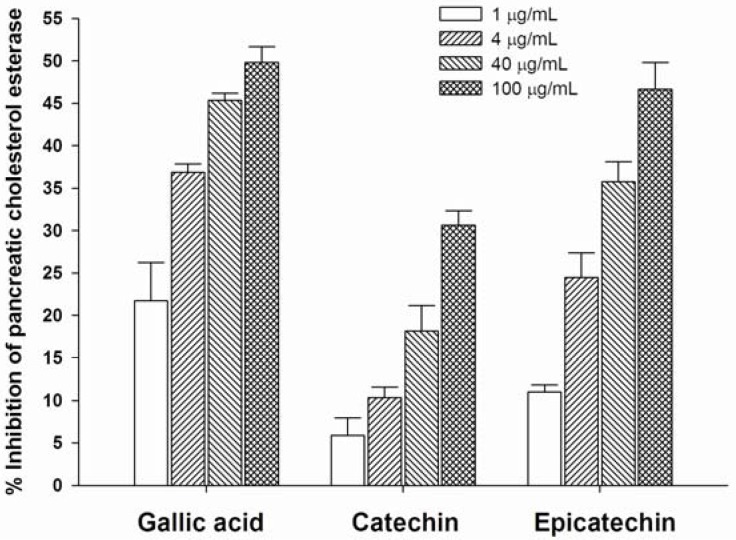
The inhibitory effect of gallic acid, catechin, and epicatechin against pancreatic cholesterol esterase. Results are expressed as mean ± S.E.M., n = 3.

As shown in Figure 1, gallic acid, catechin and epicatechin significantly inhibited cholesterol esterase in a concentration-dependent manner. However, these compounds (IC_50_ > 100 μg/mL) were less potent than simvastatin (IC_50_ = 0.08 ± 0.01 μg/mL) which was used as a pancreatic cholesterol esterase inhibitor. In general, pancreatic cholesterol esterase plays an important role in hydrolyzing dietary cholesterol esters which liberates free cholesterol in the lumen of the small intestine [13]. Furthermore, it enhances the incorporation of cholesterol into mixed micelles and aids transport of free cholesterol to the enterocyte [14]. Therefore, the inhibition of cholesterol esterase is expected to limit the absorption of dietary cholesterol, resulting in reduced cholesterol absorption. In the present study, we found that three polyphenols inhibit pancreatic cholesterol esterase which may enhance control of the bioavailability of dietary cholesterol derived from cholesterol esters and the limitation of absorption of free cholesterol into blood circulation. Our previous study has shown that grape seed extract markedly inhibits pancreatic cholesterol esterase [15]. It can be inferred that the pancreatic cholesterol esterase inhibitory activity of grape seed extract may be partly due to the effect of these three polyphenols. 

### 2.2. Bile Acid Binding

**Figure 2:**
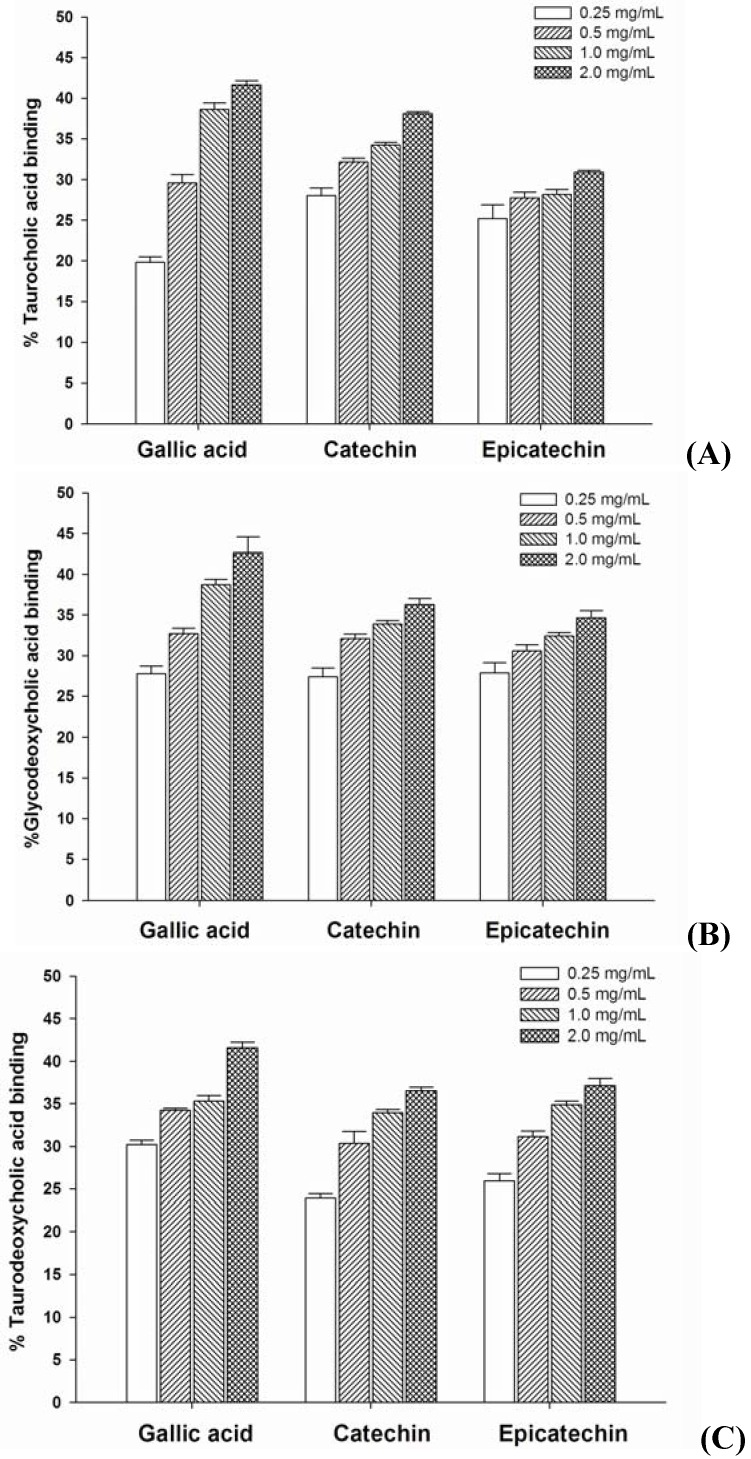
The percentage bile acid binding of gallic acid, catechin, and epicatechin. Results are expressed as mean ± S.E.M., n = 3.

The percentages of taurocholic acid and glycodeoxycholic acid binding of gallic acid, catechin, and epicatechin are shown in Figure 2A and 2B. They bound to bile acids in a concentration-dependent manner. When comparing the concentration of polyphenols (1 mg/mL), the percentage of taurocholic acid binding was: gallic acid (38.64 ± 0.77%) > catechin (34.26 ± 0.77%) > epicatechin (28.17 ± 0.61%), whereas cholestyramine bound 61.2 ± 1.27% of taurocholic acid. Considering glycodeoxycholic acid, the percentage binding was: gallic acid (38.73 ± 0.68%) > catechin (33.92 ± 0.39%) > epicatechin (32.43 ± 0.46%) and cholestyramine bound an average of 67.34 ± 5.45%. The percentage of taurodeoxycholic acid binding by gallic acid, catechin, and epicatechin is shown in Figure 2C. The percentage of taurodeoxycholic acid binding by gallic acid (35.31 ± 0.67%) was similar to catechin (33.93 ± 0.37%), and epicatechin about 34.86 ± 0.45%, respectively, whereas the percentage of taurodeoxycholic acid binding of cholestyramine (1 mg/mL) was 74.36 ± 1.13%. 

The binding of bile acids and increasing of their fecal excretion have been hypothesized as a possible mechanism for lowering plasma cholesterol levels. Cholestyramine, a bile acid sequestrant, disrupts the enterohepatic circulation of bile acids by sequestering them and preventing their reabsorption from the gut; this consequently reduces the bile acid pool. A greater amount of cholesterol is converted to bile acids to maintain a steady level in blood circulation leading to a decreased plasma cholesterol level [16]. The present study indicates that gallic acid, catechin, and epicatechin exhibit a primary bile acid-binding capacity (glycodeoxycholic acid and taurocholic acid). As in our previous report, taurocholic acid, glycodeoxycholic acid and taurodeoxycholic acid are bound by grape seed extract to a degree of 30%, 70% and 25%, respectively [15]. We suggest that the ability of grape seed extract to bind bile acids may be linked to the gallic acid, catechin, and epicatechin content. Interestingly, it has been reported that the high level of secondary bile acid is associated with increased risk of developing colorectal cancer [17]. The results also show that gallic acid, catechin, and epicatechin bind to the secondary bile acid (taurodeoxycholic acid). Therefore, a decrease in secondary bile acid concentration by these polyphenolic compounds may reduce the risk factor for developing colorectal cancer. 

### 2.3. The Solubility of Cholesterol in Micelles

**Figure 3:**
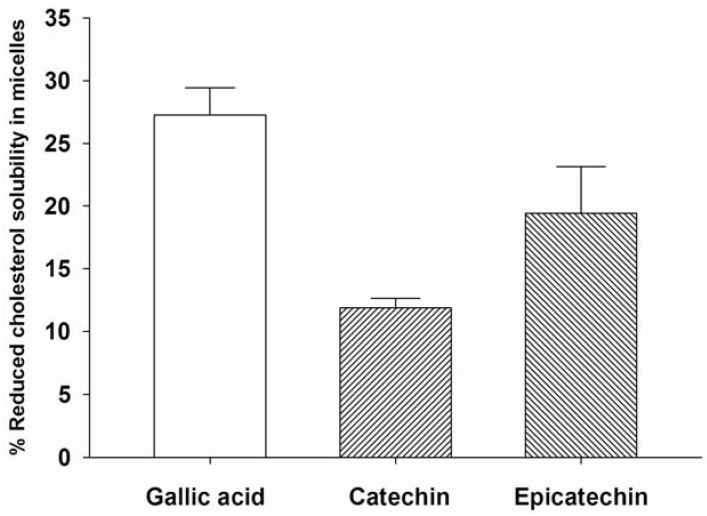
The percentage reduced cholesterol solubility in micelles of gallic acid, catechin, and epicatechin. Results are expressed as mean ± S.E.M., n = 3.

As shown in Figure 3, gallic acid, catechin, and epicatechin (0.2 mg/mL) significantly reduce the solubility of cholesterol in artificially prepared micelles by 27.26 ± 2.17%, 11.88 ± 0.75%, and 19.49 ± 3.71%, respectively, whereas cellulose (0.2 mg/mL) reduced cholesterol solubility by approximately 20.3 ± 1.34%. The principal steps in the absorption of dietary cholesterol are emulsification, hydrolysis of the ester bond by a pancreatic esterase, micellar solubilization, and absorption in the proximal jejunum [18]. It has recently been reported that the reduction of cholesterol absorption by reducing the solubility of cholesterol micellization in the intestinal lumen is a new target site of intervention for the treatment of hyperlipidemia and obesity [19]. In addition, the cholesterol-lowering effect of grape seed extract has also been reported. It has shown that the oral administration of grape seed extract with a high fat emulsion decreases blood cholesterol levels [15]. In the present study, it can be speculated that gallic acid, catechin, and epicatechin in grape seed extract may play a vital role in the suppression of cholesterol absorption by reducing solubility of cholesterol micellization. 

The reduced cholesterol solubility in micelles has also been reported for green tea epigallocatechin gallate (EGCG) [20,21]. It is suggested that EGCG interferes with the structure of micellar cholesterol, inducing the micelles to be larger and insoluble, thus inhibiting their formation. We hypothesize that the reduction of solubility of cholesterol micellization by gallic acid, catechin, and epicatechin may be associated with changes in the structure of micellar cholesterol. To prove this hypothesis, further investigation on changes in the structure of micellar cholesterol is needed.

## 3. Experimental

### 3.1. Chemical

(+)-Catechin, (–)-epicatechin, gallic acid, *p*-nitrophenylbutylrate (*p*-NPB), oleic acid, phosphatidyl-choline, glycodeoxycholic acid, taurodeoxycholic acid, taurocholic acid and porcine cholesterol esterase were purchased from Sigma-Aldrich Co. (St. Louis, MO, USA). Cholesterol test kits were purchased from HUMAN GmbH Co. (Wiesbaden, Germany). A total bile acid kit was purchased from Bio-Quant Co. (San Diego, CA, USA). All other chemical reagents used in this study were of analytical grade.

### 3.2. Pancreatic Cholesterol Esterase Assay

The pancreatic cholesterol esterase inhibition assay was performed according to a previously reported method [22]. Various concentrations of each compound were incubated with mixtures containing 5.16 mM taurocholic acid, 0.2 mM *p*-NPB in 100 mM sodium phosphate buffer, 100 mM NaCl, pH 7.0. The reaction was initiated by adding porcine pancreatic cholesterol esterase (1 μg/mL). After incubation for 5 min at 25 °C, the mixtures were measured the absorbance at 405 nm. Simvastatin was used as a positive control for this study.

### 3.3. Bile Acid Binding Assay

The bile acid binding assay was slightly modified according to a previous method [23]. Briefly, each compound was incubated with bile acid (2 mM) containing 0.1 M phosphate buffer-Saline (PBS), pH 7 at 37 °C for 90 min. The mixtures were filtered through 0.2 μm filter and frozen at −20 °C until analysis was carried out. The bile acid concentration was determined by using a bile acid analysis kit. Cholestyramine was used as a positive control in this study.

### 3.4. Cholesterol Micellization 

Artificial micelles were prepared according to a previously published method [19] with minor modifications. Briefly, the solution (2 mM cholesterol, 1 mM oleic acid, and 2.4 mM phosphatidylcholine) were dissolved in methanol and dried under nitrogen before adding 15 mM phosphate-buffered saline (PBS) containing 6.6 mM taurocholate salt, pH 7.4. The suspension was sonicated twice for 30 min using a sonicator. The micelle solution was incubated overnight at 37 °C. Each compound and equivalent PBS as control were added to the mixed micelle solution and incubated for a further 2 h at 37 °C. The solution was then centrifuged at 16,000 rpm for 20 min. The supernatant was collected for the determination of cholesterol concentration by using total cholesterol test kits. Cellulose was used as a positive control in this study.

## 4. Conclusions

Our data clearly indicate that the three major polyphenols in grape seed – gallic acid, catechin, and epicatechin – are shown to inhibit pancreatic cholesterol esterase. In particular, they also bind to bile acids, and reduce the solubility of cholesterol in micelles. The present study provides the scientific evidences for the cholesterol-lowering mechanisms of three major polyphenols present in a particular grape seed.

## References

[1] Knekt P., Kumpulainen J., Jarvinen R., Rissanen H., Heliövaara M., Reunanen A., Hakulinen T., Aromaa A. (2002). Flavonoid intake and risk of chronic diseases. Am. J. Clin. Nutr..

[2] Song Y., Manson J.E., Buring J.E., Sesso H.D., Liu S. (2005). Associations of dietary flavonoids with risk of type 2 diabetes, and markers of insulin resistance and systemic inflammation in women: A prospective study and cross-sectional analysis. J. Am. Coll. Nutr..

[3] Moreno D.A., Ilic N., Poulev A., Brasaemle D.L., Fried S.K., Raskin I. (2003). Inhibitory effects of grape seed extract on lipases. Nutrition.

[4] Terra X., Montagut G., Bustos M., Llopiz N., Ardèvol A., Bladé C., Fernández-Larrea J., Pujadas G., Salvadó J., Arola L., Blay M. (2009). Grape-seed procyanidins prevent low-grade inflammation by modulating cytokine expression in rats fed a high-fat diet. J. Nutr. Biochem..

[5] Mayer R., Stecher G., Wuerzner R., Silva R.C., Sultana T., Trojer L., Feuerstein I., Krieg C., Abel G., Popp M., Bobleter O., Bonn G.K. (2008). Proanthocyanidins: target compounds as antibacterial agents. J. Agric. Food Chem..

[6] Weber H.A., Hodges A.E., Guthrie J.R., O’Brien B.M., Robaugh D., Clark A.P., Harris R.K., Algaier J.W., Smith C.S. (2007). Comparison of proanthocyanidins in commercial antioxidants: grape seed and pine bark extracts. J. Agric. Food Chem..

[7] Yilmaz Y., Toledo R.T. (2004). Major flavonoids in grape seeds and skins: Antioxidant capacity of catechin, epicatechin, and gallic acid. J. Agric. Food Chem..

[8] Applequist W.L., Johnson H., Rottinghaus G. (2008). (+)-Catechin, (−)-Epicatechin, and Gallic Acid Content of Seeds of Hybrid Grapes Hardy in Missouri. Am. J. Enol. Vitic..

[9] Hsu C.L., Yen G.C. (2007). Effect of gallic acid on high fat diet-induced dyslipidaemia, hepatosteatosis and oxidative stress in rats. Br. J. Nutr..

[10] Simonetti P., Ciappellano S., Gardana C., Bramati L., Pietta P. (2002). Procyanidins from *Vitis vinifera* seeds: *in vivo* effects on oxidative stress. J. Agric. Food Chem..

[11] Shi J., Yu J., Pohorly J.E., Kakuda Y. (2003). Polyphenolics in grape seeds-biochemistry and functionality. J. Med. Food.

[12] Steffen Y., Schewe T., Sies H. (2005). Epicatechin protects endothelial cells against oxidized LDL and maintains NO synthase. Biochem. Biophys. Res. Commun..

[13] Brodt-Eppley J., White P., Jenkins S., Hui D.Y. (1995). Plasma cholesterol esterase level is a determinant for an atherogenic lipoprotein profile in normolipidemic human subjects. Biochim. Biophys. Acta.

[14] Myers-Payne S.C., Hui D.Y., Brockman H.L., Schroeder F. (1995). Cholesterol esterase: a cholesterol transfer protein. Biochemistry.

[15] Adisakwattana S., Moonrat J., Srichairat S., Chanasit C., Tirapongporn H., Chanathong B., Ngamukote S., Sapwarobol S., Mäkynen K. (2010). Lipid-Lowering mechanisms of grape seed extract (*Vitis vinifera* L) and its antihyperlidemic activity. J. Med. Plants Res..

[16] Insull W. (2006). Clinical utility of bile acid sequestrants in the treatment of dyslipidemia: A scientific review. South Med. J..

[17] Peterlik M. (2008). Role of bile acid secretion in human colorectal cancer. Wien Med. Wochenschr..

[18] Hui D.Y., Howles P.N. (2005). Molecular mechanisms of cholesterol absorption and transport in the intestine. Semin. Cell Dev. Biol..

[19] Kirana C., Rogers P.F., Bennett L.E., Abeywardena M.Y., Patten G.S. (2005). Naturally derived micelles for rapid *in vitro* screening of potential cholesterol-lowering bioactives. J. Agric. Food Chem..

[20] Ikeda I., Imasato Y., Sasaki E., Nakayama M., Nagao H., Takeo T., Yayabe F., Sugano M. (1992). Tea catechins decrease micellar solubility and intestinal absorption of cholesterol in rats. Biochem. Biophys. Acta.

[21] Raederstorff D.G., Schlachter M.F., Elste V., Weber P. (2003). Effect of EGCG on lipid absorption and plasma lipid levels in rats. J. Nutr. Biochem..

[22] Pietsch M., Gütschow M. (2005). Synthesis of tricyclic 1,3-oxazin-4-ones and kinetic analysis of cholesterol esterase and acetylcholinesterase inhibition. J. Med. Chem..

[23] Yoshie-Stark Y., Wäsche A. (2004). *In vitro* binding of bile acids by lupin protein isolates and their hydrolysate. Food Chem..

